# NCS-1 Deficiency Affects mRNA Levels of Genes Involved in Regulation of ATP Synthesis and Mitochondrial Stress in Highly Vulnerable *Substantia nigra* Dopaminergic Neurons

**DOI:** 10.3389/fnmol.2019.00252

**Published:** 2019-11-27

**Authors:** Carsten Simons, Julia Benkert, Nora Deuter, Christina Poetschke, Olaf Pongs, Toni Schneider, Johanna Duda, Birgit Liss

**Affiliations:** ^1^Institute of Applied Physiology, University of Ulm, Ulm, Germany; ^2^Institute of Physiology, Center for Integrative Physiology and Molecular Medicine, University of the Saarland, Homburg, Germany; ^3^Institute of Neurophysiology, University of Cologne, Cologne, Germany; ^4^New College, University of Oxford, Oxford, United Kingdom

**Keywords:** ND1, ENO2, Parkinson’s disease, mitochondrial uncoupling proteins, voltage-gated calcium channels, Cav2.3, KChip3/DREAM/Calsenilin, DJ-1/PARK7

## Abstract

Neuronal Ca^2+^ sensor proteins (NCS) transduce changes in Ca^2+^ homeostasis into altered signaling and neuronal function. NCS-1 activity has emerged as important for neuronal viability and pathophysiology. The progressive degeneration of dopaminergic (DA) neurons, particularly within the *Substantia nigra* (SN), is the hallmark of Parkinson’s disease (PD), causing its motor symptoms. The activity-related Ca^2+^ homeostasis of SN DA neurons, mitochondrial dysfunction, and metabolic stress promote neurodegeneration and PD. In contrast, NCS-1 in general has neuroprotective effects. The underlying mechanisms are unclear. We analyzed transcriptional changes in SN DA neurons upon NCS-1 loss by combining UV-laser microdissection and RT-qPCR-approaches to compare expression levels of a panel of PD and/or Ca^2+^-stress related genes from wildtype and NCS-1 KO mice. In NCS-1 KO, we detected significantly lower mRNA levels of mitochondrially coded ND1, a subunit of the respiratory chain, and of the neuron-specific enolase ENO2, a glycolytic enzyme. We also detected lower levels of the mitochondrial uncoupling proteins UCP4 and UCP5, the PARK7 gene product DJ-1, and the voltage-gated Ca^2+^ channel Cav2.3 in SN DA neurons from NCS-1 KO. Transcripts of other analyzed uncoupling proteins (UCPs), mitochondrial Ca^2+^ transporters, PARK genes, and ion channels were not altered. As Cav channels are linked to regulation of gene expression, metabolic stress and degeneration of SN DA neurons in PD, we analyzed Cav2.3 KO mice, to address if the transcriptional changes in NCS-1 KO were also present in Cav.2.3 KO, and thus probably correlated with lower Cav2.3 transcripts. However, in SN DA neurons from Cav2.3 KO mice, ND1 mRNA as well as genomic DNA levels were elevated, while ENO2, UCP4, UCP5, and DJ-1 transcript levels were not altered. In conclusion, our data indicate a possible novel function of NCS-1 in regulating gene transcription or stabilization of mRNAs in SN DA neurons. Although we do not provide functional data, our findings at the transcript level could point to impaired ATP production (lower ND1 and ENO2) and elevated metabolic stress (lower UCP4, UCP5, and DJ-1 levels) in SN DA neurons from NCS-1 KO mice. We speculate that NCS-1 is involved in stimulating ATP synthesis, while at the same time controlling mitochondrial metabolic stress, and in this way could protect SN DA neurons from degeneration.

## Introduction

Ca^2+^ signaling is important for a variety of neuronal functions, like membrane excitability, neurotransmitter release, gene transcription, and many other processes crucial for neuronal functions and viability (Berridge, 1998; Brini et al., 2014). The nature, magnitude, and location of the Ca^2+^ signal is crucial for its distinct effects (La Rovere et al., 2016). Hence, intracellular Ca^2+^ levels are tightly controlled (Gleichmann and Mattson, 2011; Heine et al., 2019). Neuronal Ca^2+^ sensor proteins (NCS) respond to changes in intracellular Ca^2+^ concentrations with conformational changes that allow them to bind diverse interaction partners, and to activate a variety of different signaling pathways (Burgoyne and Haynes, 2012; Choudhary et al., 2018; Burgoyne et al., 2019). The widely expressed neuronal Ca^2+^ sensor NCS-1 (Pongs et al., 1993) modulates e.g., voltage-gated Ca^2+^ channels (Cav) (Tsujimoto et al., 2002; Weiss et al., 2010), A-type K^+^ channels (Nakamura et al., 2001; Guo et al., 2002), that are composed of Kv4 α- and KChip3 β-subunits in SN DA neurons (Liss et al., 2001), G-protein coupled receptor kinases (GRK), and G-protein coupled dopamine D2-receptors (Kabbani et al., 2002; Pandalaneni et al., 2015), to just name a few. Changes in NCS-1 expression will alter the relation with its target proteins and were described in a variety of diseases, including schizophrenia and Parkinson’s disease, both characterized by dysfunctional dopaminergic signaling (Koh et al., 2003; Kabbani et al., 2012; Dragicevic et al., 2014; Boeckel and Ehrlich, 2018; Bandura and Feng, 2019; Catoni et al., 2019).

Parkinson’s disease (PD) is the second most common neurodegenerative disease (Schulz et al., 2016; Obeso et al., 2017). Its motor-related symptoms are caused by a progressive loss of dopaminergic (DA) neurons, particularly within the *Substantia nigra* (SN) (Damier et al., 1999; Surmeier et al., 2017b; Giguere et al., 2018). While the cause for most PD cases is still unclear, ion channel activity, activity-related Ca^2+^ homeostasis, mitochondrial dysfunction, and elevated metabolic stress constitute key interacting events in PD pathology (Duda et al., 2016; Michel et al., 2016; Surmeier et al., 2017b; Cherubini and Wade-Martins, 2018). In some familial inherited PD cases, disease-causing mutations in so-called PARK genes have been identified, most of them linked to elevated metabolic stress (van der Brug et al., 2015; Deng et al., 2018; Blauwendraat et al., 2019). SN DA neurons are particularly vulnerable to metabolic stress and other PD-stressors, due to their energetically demanding large axonal arborizations, as well as a stressful Ca^2+^ entry during action potential firing, mediated by Cav channels. This distinguishes them from the more resistant DA neurons in the ventral tegmental area (VTA) that are rarely affected in PD (Pissadaki and Bolam, 2013; Pacelli et al., 2015; Duda et al., 2016; Surmeier et al., 2017a). These activity-related, voltage-gated Ca^2+^ dynamics sustain electrical activity, ATP synthesis, and dopamine release of SN DA neurons and thus movement, but they constitute an intrinsic metabolic burden (Guzman et al., 2010, 2018; Duda et al., 2016; Zaichick et al., 2017). Moreover, the Ca^2+^ buffer capacity of calbindin negative SN DA neurons is low compared to other neurons, e.g., the resistant calbindin positive VTA DA neurons (Foehring et al., 2009; Dopeso-Reyes et al., 2014; Blesa and Vila, 2019). They rely mainly on mitochondrial-ER Ca^2+^ buffering (Cali et al., 2013; Lee et al., 2018) and on inhibitory regulatory feedback mechanisms that reduce activity related Ca^2+^ entry and associated neurodegenerative events, like Ca^2+^ dependent activation of K^+^ channels (Wolfart et al., 2001; Anderson et al., 2013; Dragicevic et al., 2014; Subramaniam et al., 2014; Iyer et al., 2017). One of these Ca^2+^ and K^+^ channel dependent feedback mechanisms operant in SN DA neurons involves NCS-1 function (Dragicevic et al., 2014; Poetschke et al., 2015; Catoni et al., 2019).

NCS-1 has emerged as particularly important in this context of activity-related Ca^2+^ stress and vulnerability of SN DA neurons in PD (Bandura and Feng, 2019; Catoni et al., 2019). In general, NCS-1 has been shown to stimulate mitochondrial function and neuronal survival promotion (Nakamura et al., 2006, 2019; Angebault et al., 2018; Boeckel and Ehrlich, 2018). Furthermore, especially in SN DA neurons, NCS-1 inhibits their stressful activity in a Ca^2+^ and Cav dependent fashion, by stimulation of inhibitory, K^+^ channel coupled dopamine D2-autoreceptors (Kabbani et al., 2002; Dragicevic et al., 2014; Robinson et al., 2017), and NCS-1 mRNA levels are elevated in remaining human SN DA neurons from *post-mortem* PD brains (Dragicevic et al., 2014).

Here, we aimed to gain insights into effects of general NCS-1 loss on gene expression in SN DA neurons. By combining UV-lasermicrodissection (UV-LMD) with retrograde tracing and quantitative PCR approaches, we examined a panel of candidate genes in SN DA neurons from NCS-1 KO mice and wildtype controls. We quantified mRNA levels of the voltage-gated Ca^2+^ channel α-subunits Cav1.3, Cav2.3, Cav3.1, the voltage- and Ca^2+^-gated A-type K^+^ channel α- and β-subunits Kv4.3 and KChip3, the mitochondrial Ca^2+^ transporters MCU, LETM1, mNCX, the mitochondrial uncoupling proteins UCP2 – UCP5, and of PARK genes that affect mitochondrial function and/or Ca^2+^ homeostasis (SNCA, DJ-1, PGC-1α, and GBA1). Furthermore, we analyzed the expression of the glycolytic enzyme neuron-specific enolase 2 (ENO2) and of the NADH-ubiquinone oxidoreductase chain 1 (ND1), a subunit of the complex I of the mitochondrial respiratory chain. We gained evidence for selective transcriptional downregulation of proteins involved in ATP synthesis (ND1, ENO2) and metabolic stress defense (UCP4, UCP5, DJ-1) in SN DA neurons from NCS-1 KO mice, that were associated with but likely not causal to lower Cav2.3 mRNA levels.

We conclude that NCS-1 (directly or indirectly) is involved in stimulating the transcription or the mRNA stability of these genes in SN DA neurons.

## Materials and Methods

### Ethical Approval

All animal procedures were approved by the German Regierungspräsidium Tübingen (AZ 35/9185.81-3TV No. 1291, Reg. No. o.147) and conducted to the guidelines of the German Tierschutzgesetz.

### Mice

All mice were bred in in-house breeding facilities at Ulm University. The NCS-1 KO is back-crossed at least 10 times into C57BL/6J, thus reaching a 99% analogy with C57BL/6J and losing the 129/SvJ original background (Ng et al., 2016). Cav2.3 KO mice were only back-crossed 4 times, leading to a 75% C57BL/6J and 25% 129/SvJ mixed background, as they do not breed well, likely due to the physiological function of Cav2.3 in mouse sperms (Wennemuth et al., 2000). SN DA neurons from NCS-1 and Cav2.3 KO mice were compared to their respective distinct +/+ wildtype background littermates. For NCS-1 KO and their WT, we analyzed juvenile mice (PN13), for Cav2.3 KO and their WT, we show results for adults (∼ PN90), as in contrast to NCS-1, Cav2.3 expression increases with post-natal maturation (Benkert et al., 2019). Data were derived from 18 NCS-1 KO and 18 NCS-1 WT mice, and from 5 Cav2.3 KO and 5 Cav2.3 WT mice. Only male mice were analyzed.

### *In vivo* Retrograde Tracing

SN DA neurons from adult mice were retrogradely labeled as described (Liss et al., 2005; Lammel et al., 2008; Krabbe et al., 2015). Red fluorescent latex retrobeads (Lumafluor) were injected unilaterally into the dorsal striatum (bregma: 0.98 mm, lateral 1.9 and 2.7 mm, ventral: -3.2 mm, 2 × 60 nl) under stereotactic control (Kopf Instruments) with a NanoFil syringe attached to a micropump (UMP3 with SYS-Micro4 Controller; World Precision Instruments) at a rate of 50 nl/min and general isoflurane anesthesia. After 7 days, mice were sacrificed and striatal injection sites were verified using TH-immunofluorescence stain (primary antibody: rabbit anti-TH, 1:1,000, catalog no. 657012, Merck group; secondary antibody: Alexa Fluor 488 goat anti-rabbit IgG, 1:1,000, catalog no. A-11034, Thermo Fisher Scientific) and Nissl stain (Neuro Trace 640/660 deep-red fluorescent Nissl stain solution, 1:1,000 in 1x PBS, catalog no. N21483, Thermo Fisher Scientific), according to the mouse brain atlas (Paxinos and Franklin, 2013) as described (Liss et al., 2005; Krabbe et al., 2015).

### Tissue Preparation, UV-Laser Microdissection (UV-LMD), and Reverse Transcription

Carried out similarly as previously described in detail (Grundemann et al., 2011; Dragicevic et al., 2014; Duda et al., 2018). Briefly, 12 μm coronal mouse brain sections were cut with a cryomicrotome CM3050 S (Leica), mounted on PEN-membrane slides (Microdissect), and fixed with an ascending ethanol series. Sections were stored in sterile Falcon tubes with silica gel at -80°C until used for UV-LMD. UV-LMD was carried out with a LMD7000 system (Leica Microsystems); laser-microdissected cells (pools of 10 SN DA neurons, each) were collected into the lid of a certified RNase free reaction tube (PCR Tubes Thinwall Clear 0.5 ml, Axygen) containing 4.7 μl lysis-buffer, and reverse transcription was performed without an RNA-isolation step by using random hexamer primers and superscript II RT enzyme (Thermo Fisher Scientific). For juvenile mice, mounted sections were stained (1 min) with a cresylviolet-ethanol solution, and SN DA neurons were identified by anatomical location, morphology and size in brightfield mode. In adult mice, fluorescence-traced SN DA neurons were identified under Y3 filter (565–610 nm, exposure time: 250 ms). The size/area of lasered cells was determined automatically after calibration by the LMD7000 software (Version 8.2.0.6739, Leica Microsystems). We ensured that lasered areas were similar for all analyzed animals/compared groups (Supplementary Figure S1 and Supplementary Table S2).

### Qualitative Multiplex Nested PCR and Quantitative Realtime PCR

All cDNA reactions were precipitated as described (Liss, 2002) and resolved in 17 μl RNase free water (5Prime, Molecular biology grade, certified RNase free). Qualitative and quantitative PCRs were carried out, essentially as described (Liss et al., 2001; Grundemann et al., 2011; Poetschke et al., 2015; Duda et al., 2018). All primers except for ND1 were spanning at least one intron to quantify only cDNA-derived signals. We performed a qualitative multiplex PCR with the GeneAmp PCR System 9700, Thermo Fisher Scientific, with an aliquot of 5 μl (30%) of each individual cDNA-pool for respective marker genes: Tyrosine hydroxylase (TH) as a marker for dopaminergic midbrain neurons, the glutamic acid decarboxylase isoforms GAD_65_ and GAD_67_ as markers for GABAergic neurons, glial fibrillary acidic protein (GFAP) as a marker for astroglia cells and calbindin_*d*__28__*k*_ (CBd28k), that is strongly expressed only in less vulnerable DA midbrain neurons. Qualitative PCR products were analyzed in a QIAxcel Advanced System (Quiagen). Only cDNA pools expressing the correct marker gene profile (i.e., TH positive, GAD, GFAP, CBd28k negative) were further analyzed via qPCR. All primer pairs and amplicon information are given in Supplementary Table S1.

Quantitative realtime PCR was performed as described (Grundemann et al., 2011; Duda et al., 2018), by using the 7900 HT Fast realtime PCR System and QuantStudio 3 System, both Thermo Fisher Scientific Thermocyclers. TaqMan^TM^ primer/probe assays were marked with a 3′ BHQ (black hole quencher) and 5′ FAM (Carboxyfluorescein). TaqMan assays were carefully established and performance was evaluated by generating standard curves, using defined amounts of cDNA (derived from midbrain tissue mRNA), over four magnitudes of 10-fold dilutions as templates, in at least three independent experiments, as described (Liss, 2002; Duda et al., 2018). All TaqMan assays and standard curve details are summarized in Table 1.

**TABLE 1:** TaqMan^®^ assay and standard curve information.

					**Standard curve data**				
**Assay ID**	**Target gene**	**Genbank accession no. (NCBI)**	**Amplicon length [bp]**	**Exon boundary**	**Threshold**	**Y-Intercept**	**Slope**	***R*^2^**	***n***
Mm00551392_m1	Cav1.3 (Cacna1d)	NM_001083616	62	38–39	0.5	45.81 ± 0.46	−3.39 ± 0.02	1.00 ± 0.00	3
Mm00494444_m1	Cav2.3 (Cacna1e)	NM_009782.3	63	43–44	0.5	43.19 ± 0.22	−3.39 ± 0.04	1.00 ± 0.00	3
Mm00486549_m1	Cav3.1 (Cacna1g)	NM_009783.3	57	9–10	0.5	42.81 ± 0.28	−3.31 ± 0.08	0.99 ± 0.01	3
Mm00498538_m1	DJ-1 (PARK7)	NM_020569.3	92	3–4	0.5 0.07^∗^	42.21 ± 0.24 41.85 ± 0.70^∗^	−3.41 ± 0.03 −3.56 ± 0.11^∗^	1.00 ± 0.00 0.99 ± 0.01^∗^	4 3^∗^
Mm00469062_m1	Eno2	NM_013509	76	7–8	0.5 0.06^∗^	41.53 ± 0.72 40.31 ± 0.21^∗^	−3.38 ± 0.06 −3.46 ± 0.04^∗^	0.99 ± 0.00 1.00 ± 0.00^∗^	3 3^∗^
Mm00484700_m1	GBA1 (Gba)	NM_008094.5	76	5–6	0.5	43.53 ± 0.55	−3.37 ± 0.07	0.99 ± 0.01	3
Mm01339777_m1	KChip3 (Kcnip3)	NM_019789.4	77	8–9	0.5	40.81 ± 0.40	−3.39 ± 0.05	1.00 ± 0.00	4
Mm00498260_m1	Kv4.3 (Kcnd3)	NM_019931.1	84	4–5	0.5	44.67 ± 0.13	−3.54 ± 0.01	1.00 ± 0.00	4
Mm00522265_m1	Letm1	NM_019694.1	74	7–8	0.5	42.22 ± 0.06	−3.37 ± 0.02	1.00 ± 0.00	3
Mm01168774_m1	MCU (Ccdc109a)	NM_001033259.4	92	4–5	0.5	43.11 ± 0.15	−3.44 ± 0.05	1.00 ± 0.00	4
Mm00519260_m1	mNCX (Slc8b1)	NM_133221.2	67	8–9	0.5	48.76 ± 0.71	−3.21 ± 0.16	0.98 ± 0.02	5
Mm00490549_m1	NCS-1	NM_019681	73	3–4	0.07^∗^	40.89 ± 0.64^∗^	−3.49 ± 0.11^∗^	1.00 ± 0.00^∗^	4^∗^
Mm04225274_s1	ND1	NC_005089_ND1.0	81	–	0.5 0.07^∗^	34.60 ± 0.44 33.23 ± 0.28^∗^	−3.44 ± 0.02 −3.51 ± 0.08^∗^	1.00 ± 0.00 1.00 ± 0.00^∗^	3 4^∗^
Mm00447181_m1	PGC-1α (Ppargc1a)	NM_008904.2	78	3–4	0.5	41.88 ± 0.11	−3.28 ± 0.02	1.00 ± 0.00	3
Mm00447331_m1	alphaSYN (Snca)	NM_001042451.2	71	2–3	0.5	42.00 ± 0.10	−3.34 ± 0.03	1.00 ± 0.00	4
Mm00627598_m1	UCP2 (Slc25a8)	NM_011671.5	89	2–3	0.5	41.86 ± 0.11	−3.21 ± 0.01	0.99 ± 0.01	3
Mm00494077_m1	UCP3 (Slc25a9)	NM_009464.3	69	5–6	0.5	47.82 ± 3.42	−3.61 ± 0.20	1.00 ± 0.00	3
Mm01277267_m1	UCP4 (Slc25a27)	NM_028711.3	76	6–7	0.5 0.06^∗^	45.20 ± 0.69 43.35 ± 0.33^∗^	−3.55 ± 0.12 3.65 ± 0.09^∗^	1.00 ± 0.00 0.99 ± 0.01^∗^	4 3^∗^
Mm00488302_m1	UCP5 (Slc25a14)	NM_001290703/4.1	81	9–10	0.5 0.06^∗^	44.66 ± 0.10 44.21 ± 0.09^∗^	−3.28 ± 0.03 −3.55 ± 0.02^∗^	0.99 ± 0.01 0.99 ± 0.00^∗^	4 3^∗^

*Threshold, Y-Intercept (± SD), slope (± SD), R^2^ (± SD) were determined using serial dilutions over four magnitudes of cDNA as templates, derived from mouse midbrain tissue of juvenile NCS-1 WT mice (^∗^Cav2.3 WT-derived).*

### Isolation of Genomic DNA From Laser-Microdissected Neurons and qPCR Quantification of Genomic ND1

Laser-microdissected pools of 10 SN DA neurons as well as single neurons were harvested into the lid of a reaction tube (PCR Tubes Thinwall Clear 0.5 ml, Axygen), containing 15 μl ATL-buffer (tissue lysis buffer, Qiagen). The QiaAmp DNA Micro-Kit (Qiagen) was used and the manufacturer protocol was adapted as described for genomic DNA isolation (Muhling et al., 2014). Genomic DNA was eluted in 30 μl water and 5 μl were used for ND1 qPCR. Mitochondrial genome copy number was estimated by quantifying the mitochondrially coded NADH-ubiquinone oxidoreductase chain I (ND1) from genomic DNA via qPCR, as described (Bender et al., 2006; Muhling et al., 2014). We quantified genomic ND1 levels in single SN DA neurons as well as in pools of 10 neurons (Supplementary Table S2 and Supplementary Figure S2). As we detected no significant differences in mean copy-number per individual SN DA neuron between both approaches, respective data sets were pooled.

### qPCR Data Analysis and Statistics

For qPCR data analysis as well as for graphical representation, the SDS 2.4 software (Thermo Fisher Scientific), the QuantStudio^TM^ Design and Analysis Software (Thermo Fisher Scientific) and GraphPad Prism 6 (GraphPad Software Inc.) were used. The cDNA-amount per cell in relation to the utilized standard was calculated as described (Grundemann et al., 2011; Schlaudraff et al., 2014; Duda et al., 2018), according to the following formula:

$$\mathrm{cDNA}\,\mathrm{amount}\,\mathrm{per}\,\mathrm{cell}=\frac{S^{\left[\left(C\,t-Y_{I\,n\,t\,e\,r\,c\,e\,p\,t}\right)/s\,l\,o\,p\,e\right]}}{N\,o_{c\,e\,l\,l\,s}\times c\,D\,N\,A\,f\,r\,a\,c\,t\,i\,o\,n}$$

*S* stands for the serial dilution factor of the standard curve (in our case 10 for serial dilution steps of 10), *No*_*cells*_ corresponds to the number of harvested neurons per cDNA sample (here 10), and cDNA fraction refers to the fraction of the cDNA (or respectively genomic ND1 DNA) reaction product, used as a template in qPCR reactions (cDNA: 1.5/17 for ND1 and ENO2; 5/17 for all other analyzed genes. Genomic DNA: 5/30 for ND1). The *Y*_*Intercept*_ unit magnitude corresponds to the respective standard utilized (e.g., pg equivalents of standard cDNA, derived from midbrain tissue mRNA). To facilitate comparison, cDNA amounts were calculated with a *Y*_*Intercept*_ of 45.00 for all genes. Expression data are given as mean ± SD with and without normalization to the neuronal size per microdissected area (by dividing respective expression values to the corresponding area of the individual microdissected neurons). Note that the mitochondrially encoded ND1 gene contains no intron, thus the RT-qPCR results reflect number of cDNA and genomic ND1 molecules per sample.

Outlier tests were performed in GraphPad Prism 6 and outliers were removed according to ROUT-outlier test. For statistical analysis, the non-parametric Mann-Whitney U (MWU) test was used. Significant differences are marked by asterisks (^∗^*p* ≤ 0.05; ^∗∗^*p* < 0.01; ^∗∗∗^*p* < 0.001, and ^****^p < 0.0001).

## Results

**FIGURE 1:**
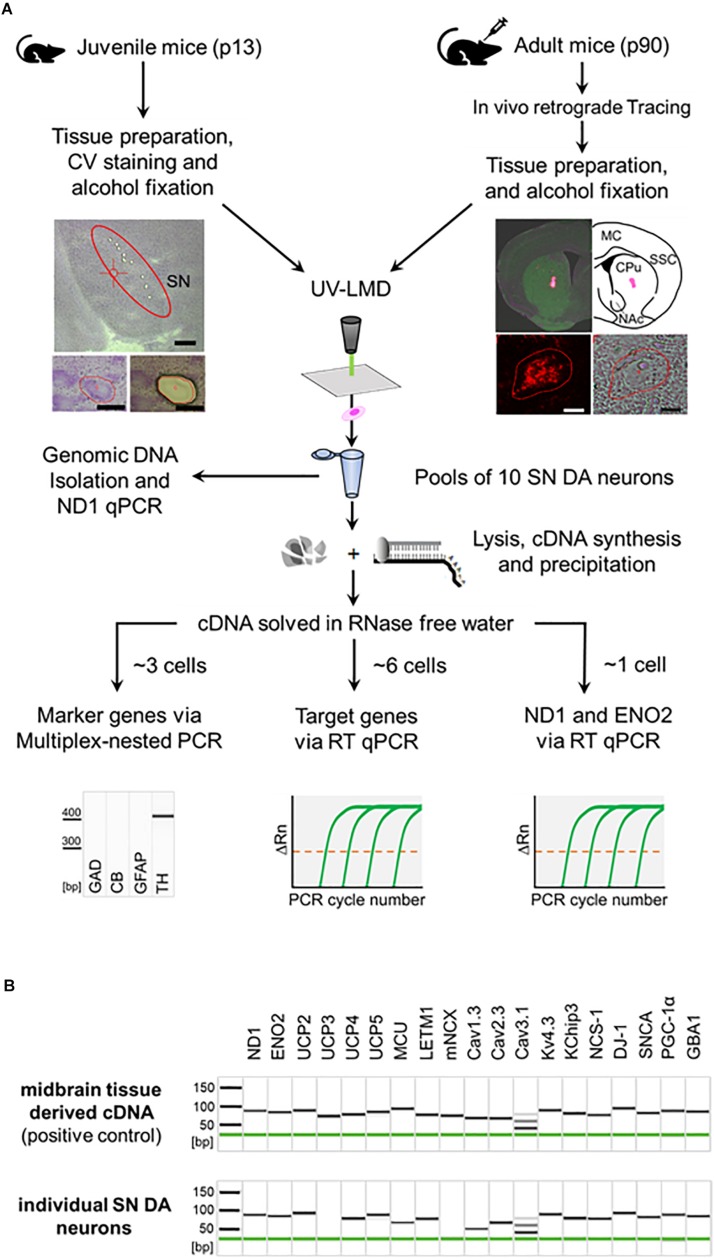
General workflow illustrating procedure for combined UV-LMD and RT-qPCR based mRNA and genomic DNA analysis for individual mouse SN DA neurons. **(A)** Coronal cryo-sections from juvenile (PN13) mice were stained with cresylviolet (CV) and ethanol-fixed. SN of adult mice (PN90) was *in vivo* retrogradely traced and coronal cryo-sections were not CV-stained but only ethanol-fixed. SN DA neurons were isolated via UV-LMD into a sterile reaction tube. Subsequently, either genomic DNA was isolated from each sample for qPCR-based quantification of genomic ND1, or a combined lysis and cDNA synthesis protocol was applied, followed by cDNA precipitation for qPCR-based mRNA quantification. A fraction of each cDNA-pool was used for qualitative multiplex-nested marker gene PCR. Note that only samples expressing the correct marker gene profile (TH positive, GAD, CB, GFAP negative) were further analyzed via TaqMan^TM^ qPCR, as indicated. GAD, L-glutamate decarboxylase; CB, calbindin_*d*__28__*k*_; GFAP, glial fibrillary acidic protein; TH, tyrosine hydroxylase. Left photographs: Upper: Overview of a juvenile CV-stained wildtype mouse coronal section after UV-LMD of 10 SN DA neurons. The Substantia nigra (SN) is highlighted. Scale bar: 500 μm. Lower: An exemplary juvenile SN DA neuron before (left) and after (right) UV-LMD. Scale bars: 30 μm. Right photographs: Upper: View of an adult *in vivo* traced injection site (i.e., dorsal striatum), next to an illustration of the respective brain section according to the mouse brain atlas (Paxinos and Franklin, 2013). MC, motor cortex; SSC, somatosensory cortex; CPu, Caudate putamen; NAc, Nucleus Accumbens. Lower: Traced SN DA neuron in fluorescence (left) and brightfield mode (right). Scale bars: 10 μm. **(B)** Upper: Gel image after capillary electrophoresis of RT-PCR products indicates that all presumed mRNAs are expressed in standard cDNA, derived from PN13 C57BL/6J mouse midbrain tissue in a 1:10^4^ dilution (positive control). Lower: Gel image after capillary electrophoresis of RT-PCR products of an individual SN DA neuron from a WT mouse indicates that all genes analyzed in this study, except for UCP3 and mNCX, are regularly expressed at the mRNA level in individual TH positive SN neurons from WT mice. Note, that we detected positive signals for mNCX only in ∼22% (2 of 9) and signals for UCP2 only in ∼50% (8 of 15) of analyzed WT SN DA neurons.

To study possible transcriptional changes in vulnerable SN DA neurons upon NCS-1 loss, we quantified mRNA levels of PD and/or Ca^2+^ stress-related genes. To analyze relative mRNA expression levels with best possible resolution, we used our well-established single cell UV-LMD RT-qPCR protocol, and optimized TaqMan primer/probe assays. Figure 1A summarizes the general work-flow, Table 1 and Supplementary Table S1 summarize details of all utilized PCR primer/probe assays. As illustrated in Figure 1B, all analyzed mRNAs are readily detected in wildtype mouse midbrain tissue, including NCS-1. This was also the case in individual laser-microdissected SN DA neurons from wildtype mice for most genes, with the following exceptions: the mitochondrial uncoupling protein UCP2 was detected only in about 50% (*n* = 8 of 15), the mitochondrial Ca^2+^ exchanger mNCX only in about 22% (*n* = 2 of 9) of SN DA neurons, while UCP3 was not detected at all, in accordance with previous work (Andrews et al., 2005; Liss et al., 2005; Hoang et al., 2012). Hence, we focused on comparing mRNA levels of ND1 and ENO2 proteins, the mitochondrial Ca^2+^ transporters MCU and LETM1, the mitochondrial uncoupling proteins UCP2, 4, and 5, the PARK genes DJ-1, SNCA, PGC-1α, and GBA1, and the ion channels Cav1.3, Cav2.3, Cav3.1, and Kv4.3/KChip3, in SN neurons that were positive for tyrosine hydroxylase, ND1 and ENO2, while negative for calbindin_*d*__28__*k*_, GAD_65__/__67_, and GFAP. RT-qPCR data were normalized to cell sizes, derived from laser-microdissected areas. Mean cell sizes/lasered areas were similar for all compared groups (Supplementary Figure S1 and Supplementary Table S2).

**FIGURE 2:**
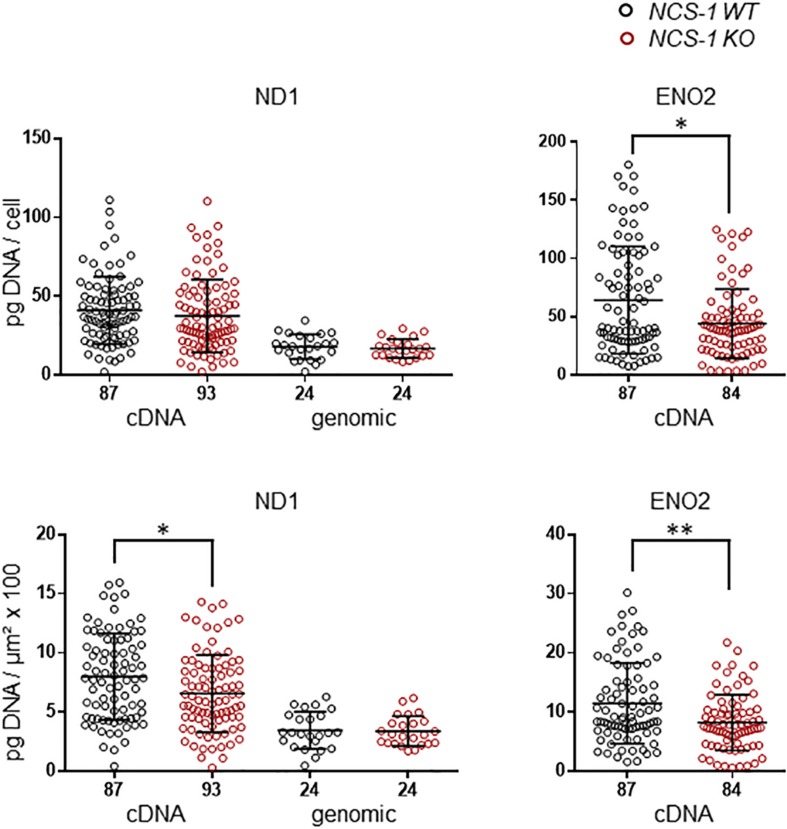
Lower relative cDNA levels of ND1 and ENO2 in SN DA neurons from NCS-1 KO mice compared to WT. Left: qPCR-derived relative cDNA and genomic DNA levels of mitochondrially coded NADH-ubiquinone oxidoreductase chain 1 (ND1) in individual TH positive SN DA neurons from juvenile NCS-1 KO and WT mice, relative to respective tissue cDNA derived standard curves (in pg/cell, upper), and in addition normalized to specific laser-microdissected neuron sizes (in μm^2^ × 100, lower). Note that ND1 cDNA levels reflect cDNA and also genomic DNA-derived signals, as the mitochondrially encoded ND1 gene contains no intron, but genomic ND1 levels alone are similar between WT and KO. Right: Similar qPCR-derived relative cDNA levels for the same samples as in (A) for the neuron specific enolase (ENO2). Data are given as scatter plots with mean ± SD. Significant differences are indicated according to Mann–Whitney U-tests and marked with asterisks (^∗^*p* ≤ 0.05; ^∗∗^*p* < 0.01). Numbers of analyzed individual SN DA neuron-derived cDNA samples (n) are given on the x-axis. All data and statistics detailed in Supplementary Figure S2 and Supplementary Table S2.

We first analyzed ND1 and ENO2 mRNA, as markers for mitochondrial and glycolytic ATP synthesis, respectively in SN DA neurons from NCS-1 KO and WT mice. Both mRNAs were readily detected in all samples, but levels for both genes were about 20% lower in NCS-1 KO (Figure 2, Table 2 and Supplementary Table S2; ND1: *n* = 87 for WT, *n* = 93 for KO, KO/WT = 0.82, *p* = 0.0152; ENO2: *n* = 87 for WT, *n* = 84 for KO, KO/WT = 0.72, *p* = 0.0034). As the mitochondrially coded ND1 gene is intronless, the RT-qPCR results reflect the amounts of cDNA and genomic DNA. To address if the lower detected mRNA levels are rather caused by lower numbers of mitochondrial genomes than by lower number of ND1 transcripts, we compared genomic ND1 levels in SN DA neurons from WT and NCS-1 KO mice (Figure 2, Table 2, and Supplementary Table S2). As genomic ND1 levels were similar, this argues for lower ND1 transcription or lower ND1 mRNA stability, but similar numbers of mitochondrial genomes in SN DA neurons from NCS-1 KO mice.

**TABLE 2:** Relative mRNA levels in SN DA neurons of either NCS-1 KO or Cav2.3 KO, normalized to individual cell sizes, and to respective WT data.

	**NCS-1 WT**						**NCS-1 KO**						
	**Mean**	** ± SEM**	**±SD**	**Median**	***n***	***N***	**Mean**	** ± SEM**	**±SD**	**Median**	***n***	***N***	***p*-value**

ND1 cDNA	1.00	0.05	0.46	0.98	87 (88)	18	0.82	0.04	0.41	0.75	93 (94)	18	0.0152^∗^
ND1 genom.	1.00	0.09	0.45	0.94	24 (25)	3	0.98	0.07	0.37	0.90	24 (25)	3	0.8182
ENO2	1.00	0.07	0.59	0.80	87 (88)	18	0.72	0.05	0.41	0.65	84 (94)	18	0.0034^∗^
UCP2	1.00	0.33	0.93	0.74	8 (15)	4	0.73	0.53	1.30	0.04	6 (15)	4	0.5728
UCP4	1.00	0.10	0.31	1.00	9 (9)	3	0.44	0.08	0.25	0.43	10 (10)	3	0.0003^∗^
UCP5	1.00	0.24	0.73	0.71	9 (9)	3	0.42	0.06	0.18	0.47	9 (10)	3	0.0142^∗^
MCU	1.00	0.25	0.74	0.69	9 (10)	3	0.91	0.26	0.68	0.69	7 (10)	3	0.9182
LETM1	1.00	0.17	0.49	0.92	8 (9)	4	0.68	0.13	0.40	0.58	9 (9)	4	0.0927
DJ-1	1.00	0.15	0.52	0.76	13 (13)	6	0.45	0.06	0.19	0.45	11 (14)	6	0.0129^∗^
SNCA	1.00	0.16	0.45	0.92	8 (9)	3	0.91	0.15	0.41	0.70	7 (8)	4	0.4634
PGC-1α	1.00	0.22	0.69	0.82	10 (10)	4	1.00	0.32	0.86	0.85	7 (8)	4	0.8868
GBA1	1.00	0.10	0.31	1.07	9 (10)	4	0.78	0.16	0.45	0.68	8 (8)	4	0.1996
Cav1.3	1.00	0.16	0.48	0.88	9 (10)	4	0.82	0.15	0.48	0.73	10 (10)	3	0.3562
Cav2.3^#^	1.00	0.09	0.39	0.88	20 (20)	5	0.53	0.06	0.28	0.51	20 (20)	4	< 0.0001^∗^
Cav3.1	1.00	0.13	0.40	1.03	10 (10)	4	0.82	0.16	0.50	0.56	10 (10)	4	0.4359
Kv4.3	1.00	0.15	0.46	0.90	10 (10)	3	1.11	0.29	0.86	0.84	9 (10)	3	0.9682
KChip3	1.00	0.16	0.51	0.90	10 (10)	3	1.06	0.24	0.75	0.74	10 (10)	3	0.8534

	**Cav2.3 WT**						**Cav2.3 KO**						
	**Mean**	**± SEM**	**±SD**	**Median**	***n***	***N***	**Mean**	**± SEM**	**±SD**	**Median**	***n***	***N***	***p*-value**
ND1 cDNA	1.00	0.04	0.26	0.96	42 (43)	5	1.14	0.04	0.27	1.07	44 (44)	5	0.0062^∗^
ND1 genom.	1.00	0.06	0.22	1.02	15 (20)	2	1.24	0.11	0.49	1.22	19 (20)	3	0.0153^∗^
ENO2	1.00	0.06	0.36	0.97	42 (43)	5	1.06	0.07	0.44	1.07	44 (44)	5	0.5330
DJ-1	1.00	0.09	0.30	0.89	10 (10)	3	0.94	0.16	0.50	0.85	10 (10)	3	0.6305
UCP4	1.00	0.10	0.33	1.02	10 (10)	3	1.28	0.21	0.67	1.30	10 (10)	3	0.2799
UCP5	1.00	0.13	0.54	0.90	16 (16)	4	1.14	0.12	0.49	1.04	16 (16)	4	0.3414

**FIGURE 3:**
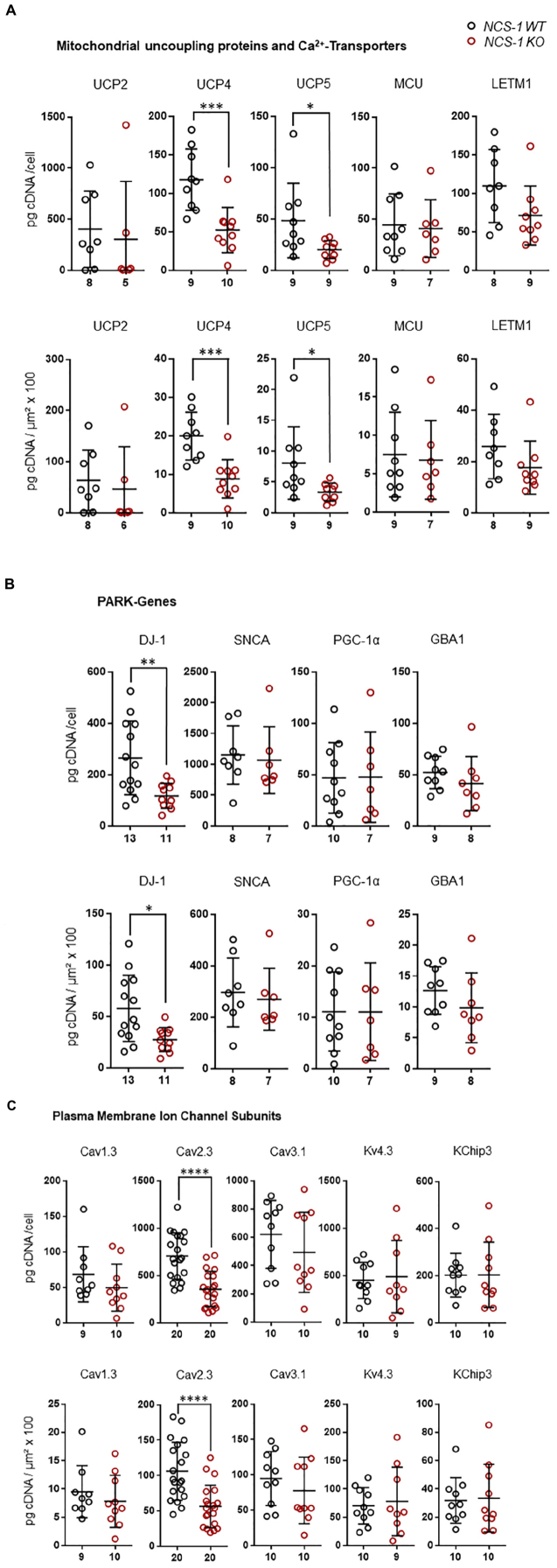
Lower mRNA levels selectively of UCP4, UCP5, DJ-1, and Cav2.3 in SN DA neurons from NCS-1 KO mice compared to WT. Relative RT-qPCR-derived data from individual TH positive SN neurons from juvenile NCS-1 KO and WT mice **(A)** for mitochondrial uncoupling proteins UCP2, UCP4, UCP5 and mitochondrial Ca^2+^ transporters MCU, and LETM1, **(B)** for the PD-associated PARK genes DJ-1, SNCA, PGC-1α, GBA1, and **(C)** for the voltage-gated ion channel pore forming α-subunits Cav1.3, Cav2.3, Cav3.2, and Kv4.3, as well as for KChip3. Data are given relative to respective tissue cDNA-derived standard curves (in pg/cell, upper), and normalized to specific laser-microdissected neuron sizes (in μm^2^ × 100, lower), as scatter plots with mean ± SD. Cav2.3 data modified from Benkert et al. (2019). Significant differences are indicated according to Mann–Whitney U-tests and marked with asterisks (^∗^*p* ≤ 0.05; ^∗∗^*p* < 0.01; ^∗∗∗^*p* < 0.001 and ^****^p < 0.0001). Numbers of analyzed individual SN DA neuron-derived cDNA samples (n) are given on the x-axis. All data and statistics detailed in Supplementary Table S3.

**FIGURE 4:**
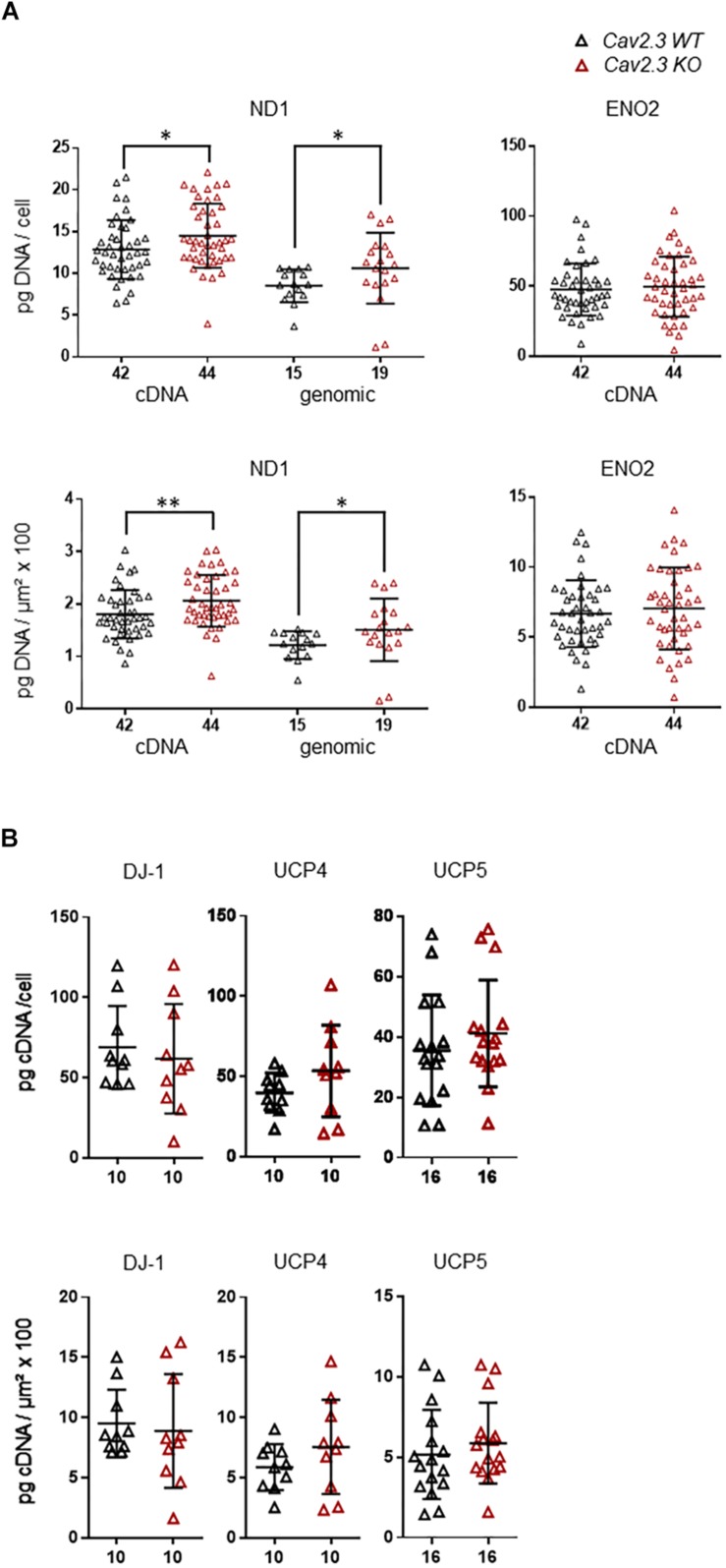
Higher expression levels selectively of ND1 cDNA and genomic DNA in SN DA neurons from Cav2.3 KO mice compared to WT. **(A)** Left: qPCR-derived relative cDNA and genomic DNA levels of mitochondrially coded NADH-ubiquinone oxidoreductase chain 1 (ND1) in individual TH positive SN DA neurons from adult Cav2.3 WT and KO mice, relative to respective tissue cDNA derived standard curves (in pg/cell, upper), or normalized to specific laser-microdissected neuron sizes (in μm^2^ × 100, lower). Note that ND1 cDNA levels reflect cDNA + genomic DNA-derived signals, as the ND1 gene contains no intron, and cDNA as well as genomic ND1 levels are elevated in the KO. Right: Similar qPCR-derived relative cDNA levels for the same samples as in (**A**, left) for the neuron-specific enolase (ENO2). **(B)** Relative RT-qPCR-derived data from individual TH positive SN neurons from Cav2.3 KO and WT mice for the genes as indicated (displaying lower mRNA levels in SN DA neurons from NCS-1 KO mice). All data are given as scatter plots with mean ± SD. Significant differences are indicated according to Mann–Whitney U-tests and marked with asterisks (^∗^*p* ≤ 0.05; ^∗∗^*p* < 0.01). Numbers of analyzed individual SN DA neuron-derived cDNA samples (n) are given on the x-axis. All data and statistics detailed in Supplementary Figure S2 and Supplementary Tables S2, S4.

**Data and statistics for graphs shown in Figures 2, 3A–C, 4A,B. Data are normalized to lasered cell sizes and to WT, thus mean KO-values directly display fold-differences in relation to respective WT. (n) indicates the number of detected signals, from all tested TH positive SN neuron-derived cDNA samples (given in brackets), derived from (N) mice. (^#^) data modified from Benkert et al. (2019). P-values according to Mann–Whitney U-tests, significant differences are marked with (^∗^). Note that ND1 cDNA levels reflect cDNA + genomic DNA-derived signals, as the ND1 gene contains no intron.**

Furthermore, we detected about 60% lower levels of the mitochondrial uncoupling proteins UCP4 and UCP5, while those of UCP2, MCU and LETM1 were not changed (Figure 3A, Table 2, and Supplementary Table S3; UCP4: *n* = 9 for WT, *n* = 10 for KO, KO/WT = 0.44, *p* = 0.0003; UCP5: *n* = 9 for WT, *n* = 9 for KO, KO/WT = 0.42, *p* = 0.0142). For mNCX, we detected signals only in 2 out of 9 WT samples and in none of the 10 NCS-1 KO samples (Supplementary Table S3), thus a statistical comparison is not meaningful. Similar as for UCP4 and UCP5, we detected about 55% lower mRNA levels of the PARK7 gene product, the deglycase DJ-1 (Figure 3B, Table 2, and Supplementary Table S3; *n* = 13 for WT, *n* = 11 for KO, KO/WT = 0.45, *p* = 0.0129). In contrast, mRNA levels of all other analyzed PD-related genes (SNCA, PGC-1α, and GBA1) were similar in SN DA neurons from NCS-1 KO and WT mice (Figure 3B, Table 2, and Supplementary Table S3). mRNA levels of Cav2.3 R-type channel α-subunits were also about 50% lower (*n* = 20 for WT, *n* = 20 for KO, KO/WT = 0.53, *p* < 0.0001) in SN DA neurons from NCS-1 KO mice. In contrast, those of Cav1.3 and of Cav3.1 were not changed. Likewise, transcript levels of Ca^2+^ sensing KChip3 and of Kv4.3 A-type K^+^ channel subunits were also similar in NCS-1 KO and WT mice (Figure 3C, Table 2, and Supplementary Table S3).

In summary, we detected at the transcriptional level an orchestrated downregulation, of ND1 and ENO2, both involved in ATP synthesis, of the mitochondrial uncoupling proteins UCP4 and UCP5, the PARK7 gene DJ-1, and of Cav2.3 in SN DA neurons from NCS-1 KO mice, compared to WT.

Cav channels can regulate gene expression (Gomez-Ospina et al., 2006; Barbado et al., 2009; Pinato et al., 2009), and in SN DA neurons Cav activity contributes to elevated metabolic stress and to their degeneration in PD (Surmeier et al., 2017a; Guzman et al., 2018; Tabata et al., 2018). To address if the lower mRNA levels of ND1, ENO2, UCP4, and 5, and DJ-1 in SN DA neurons from NCS-1 KO mice are possibly secondary to the lower Cav2.3 levels in NCS-1 KO, we analyzed the expression of these genes in SN DA neurons from Cav2.3 KO mice and respective wildtype controls. In SN DA neurons from Cav2.3 KO, ND1 mRNA as well as genomic DNA levels were about 15 and 25% higher compared to those of WT mice, respectively, pointing to more mitochondrial genomes in SN DA neurons from Cav2.3 KO (Figure 4A, Table 2, and Supplementary Table S2; ND1 mRNA + genomic DNA: *n* = 42 for WT, *n* = 44 for KO, KO/WT = 1.14, *p* = 0.0062; genomic DNA: *n* = 15 for WT, *n* = 19 for WT, KO/WT = 1.24, *p* = 0.0153). For all other tested mRNAs, levels were similar between WT and Cav2.3 KO (ENO2: Figure 4A, Table 2, and Supplementary Table S2; UCP4 and5, DJ-1: Figure 4B, Table 2, and Supplementary Table S4).

**FIGURE 5:**
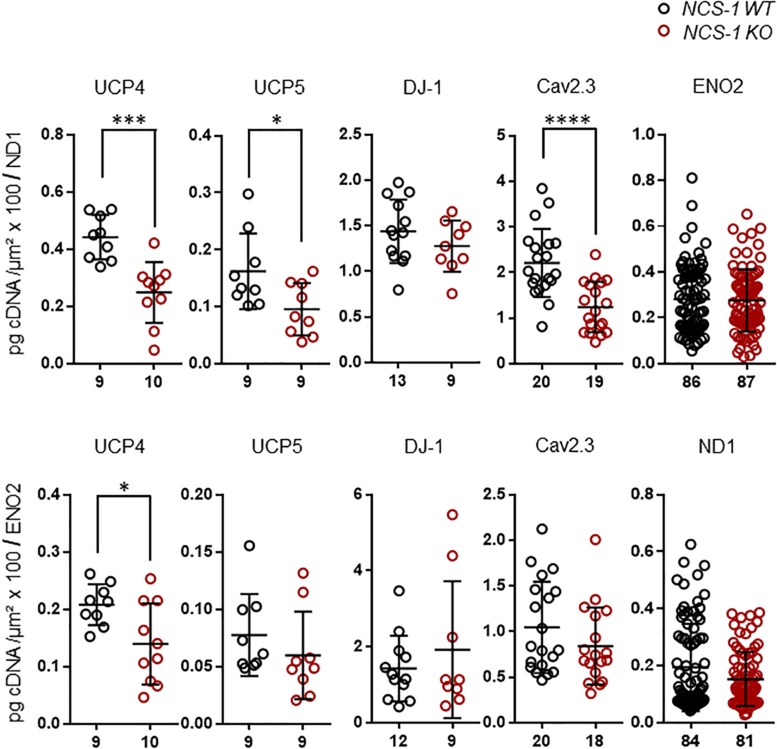
RT-qPCR data for genes with altered expression in SN DA neurons from NCS-1 KO, in relation to ND1 and ENO2 mRNA expression for the same samples. Relative mRNA expression data for NCS-1 WT and KO, normalized to cell sizes, from Figures 2, 3, plotted in relation to that of ND1 and ENO2, as indicated. Data are given as scatter plots with mean ± SD. Significant differences are indicated according to Mann–Whitney U-tests and marked with asterisks (^∗^*p* ≤ 0.05; ^∗∗∗^*p* < 0.001, and ^****^p < 0.0001). Numbers of analyzed individual SN DA neuron-derived cDNA samples (n) are given on the x-axis. All data and statistics detailed in Supplementary Table S5.

Finally, we plotted the detected transcript levels for UCP4, UCP5, DJ-1, and Cav2.3 for NCS-1 KO and WT to that of ND1 and ENO2, determined in the same samples, to assess if the detected changes in SN DA neurons from NCS-1 KO mice were correlated with each other. Lower UCP4, UCP5 and Cav2.3 mRNA levels in NCS-1 KO were not correlated to that of ND1, as they were still significantly lower in KO when compared to WT in relation to ND1 mRNA levels, while in relation to ENO2, only UCP4 mRNA was still significantly lower in NCS-1 KO (Figure 5, Table 3, and Supplementary Table S5).

**TABLE 3:** Relative mRNA levels in SN DA neurons of NCS-1 KO and WT, normalized to cell sizes, in respect to that of ND1 or ENO2.

	**NCS-1 WT**						**NCS-1 KO**						
	**Mean**	** ± SEM**	**±SD**	**Median**	***n***	***N***	**Mean**	** ± SEM**	**±SD**	**Median**	***n***	***N***	***p*-value**
	**qPCR results, normalized to neuron size, ND1 and WT**												
	
UCP4	1.00	0.06	0.18	1.02	9 (9)	3	0.60	0.08	0.24	0.63	10 (10)	3	0.0003^∗^
UCP5	1.00	0.14	0.41	0.82	9 (9)	3	0.59	0.09	0.28	0.51	9 (10)	3	0.0503^∗^
DJ-1	1.00	0.07	0.24	0.99	13 (13)	6	0.89	0.07	0.20	0.89	9 (14)	6	0.2921
Cav2.3	1.00	0.08	0.34	0.91	20 (20)	5	0.56	0.06	0.25	0.52	19 (20)	4	< 0.0001^∗^
ENO2	1.00	0.06	0.53	0.94	86 (88)	18	0.98	0.05	0.49	0.98	87 (94)	18	0.9722
	
	**qPCR results, normalized to neuron size, ENO2 and WT**												
	
UCP4	1.00	0.06	0.17	1.03	9 (9)	3	0.67	0.11	0.34	0.61	10 (10)	3	0.0350^∗^
UCP5	1.00	0.15	0.46	0.79	9 (9)	3	0.77	0.16	0.49	0.63	9 (10)	3	0.1615
DJ-1	1.00	0.17	0.60	0.85	12 (13)	6	1.35	0.42	1.26	0.79	9 (14)	6	0.9170
Cav2.3	1.00	0.11	0.48	0.78	20 (20)	5	0.80	0.10	0.40	0.68	18 (20)	4	0.1957
ND1	1.00	0.09	0.79	0.57	84 (88)	18	0.79	0.05	0.49	0.59	81 (94)	18	0.5682

**Data and statistics for graphs shown in Figure 5. Data are normalized to lasered cell sizes, to ND1 or ENO2 and to WT, thus mean KO-values directly display fold-differences in relation to respective WTs. (n) indicates the number of detected signals, from all tested TH positive SN DA neuron-derived cDNA-samples (given in brackets), derived from (N) mice. P-values according to Mann–Whitney U-tests, significant differences are marked with (^∗^).**

## Discussion

**FIGURE 6:**
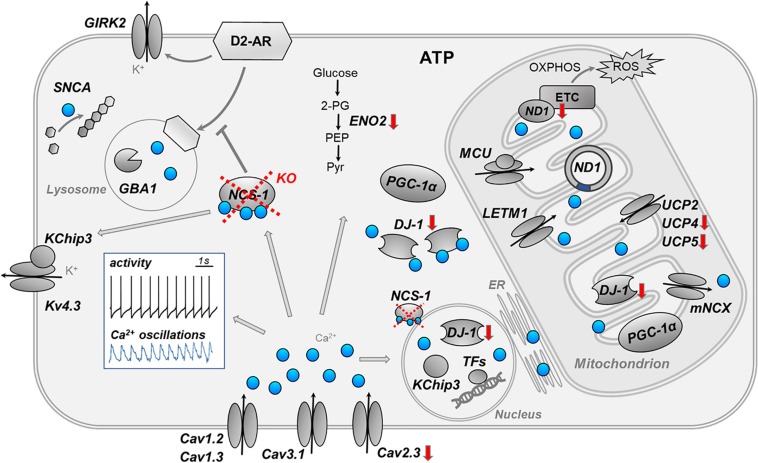
Summary cartoon of detected gene expression changes in SN DA neuron from NCS-1 KO mice. The cartoon summarizes the main findings and conclusions of this study and illustrates the complex activity- and compartment-dependent roles of Ca^2+^ signaling in vulnerable SN DA neurons. The white insert-box illustrates the autonomous pacemaker activity (black trace) and the associated Ca^2+^ transients (blue trace) of SN DA neurons (perforated patch clamp and Calcium-imaging experiment, adapted from Liss and Striessnig (2019). Loss of NCS-1 is indicated by red cross, red arrows indicate transcriptional changes in NCS-1 KO mice. Ca^2+^ (blue dots) has a variety of different physiological and potentially detrimental functions in distinct compartments of SN DA neurons, like stimulating ATP synthesis enzymes, controlling gene expression, and triggering apoptosis. Ca^2+^ can indirectly inhibit electrical activity of SN DA neurons by stimulating NCS-1/D2-AR or Kv4.3/KChip3 activity. Similar as NCS-1, KChip3 (also known as DREAM or Calsenilin) is not only a β-subunit of voltage-gated Kv4.3 K^+^ channels, but it can also translocate from the plasma-membrane to the nucleus, where it controls Ca^2+^ dependent enzymes or gene expression. For further details, see text. ATP, adenosine triphosphate; Cav, voltage-gated Ca^2+^ channel; DJ-1, protein deglycase; D2-AR, dopamine D2 autoreceptor; ENO2, neuron specific enolase; ER, endoplasmic reticulum; ETC, electron transfer chain; GBA1, glucocerebrosidase 1; GIRK2, G protein-coupled inwardly rectifying potassium channel; KChip3, Calsenilin (also known as DREAM); KO, knockout; Kv4.3, voltage-gated potassium channel subfamily D member; LETM1, leucine zipper EF-hand containing transmembrane protein 1; MCU, mitochondrial Ca^2+^ uniporter (pore-forming subunit); mNCX, mitochondrial sodium-calcium exchanger; NCS-1, neuronal calcium sensor 1; ND1, NADH-ubiquinone oxidoreductase chain 1; OXPHOS, oxidative phosphorylation; PEP, phosphoenolpyruvate; PGC-1α, peroxisome proliferator-activated receptor gamma coactivator 1-alpha; Pyr, pyruvate; ROS, reactive oxygen species; SNCA, alpha-synuclein gene; TFs, transcription factors; UCP, uncoupling protein; 2-PG, 2-phosphoglycerate.

Here, we addressed transcriptional changes in SN DA neurons, caused by the loss of NCS-1. We combined UV-LMD with RT-qPCR approaches to analyze mRNA levels in individual SN DA neurons of NCS-1 KO mice, compared to their respective wildtype. Figure 6 summarizes the main findings and conclusion of this study and illustrates complex Ca^2+^ signaling in SN DA neurons.

### NCS-1 – A Regulator of Gene Transcription in SN DA Neurons?

The finding that ND1, ENO2, UCP4, UCP5, DJ-1, and Cav2.3 transcripts are significantly lower in SN DA neurons from NCS-1 KO mice, indicates that NCS-1 is involved in regulating the transcription of these genes or the stability of their mRNAs in these neurons.

Ca^2+^ dependent regulation of gene expression in general, and in particular of genes involved in Ca^2+^ homeostasis and metabolic stress, is well described (Berridge et al., 2000; West et al., 2001; Greer and Greenberg, 2008; Naranjo and Mellstrom, 2012). For instance, a Ca^2+^ dependent regulation by calcineurin is described for NCS-1 gene expression itself (Hamasaki-Katagiri and Ames, 2010) that is stimulated by the neurotrophic factor GDNF (Nakamura et al., 2019). How exactly NCS-1 in turn could regulate gene expression remains less clear. A role of NCS-1 for controlling activity-related nuclear Ca^2+^ levels is reported for cardiomyocytes (Nakao et al., 2015). NCS-1 is present in the nucleus or in perinuclear regions (Burgoyne, 2007; Nakao et al., 2015), its nuclear targeting is Ca^2+^ dependent (O’Callaghan et al., 2002), and recent evidence suggests that NCS-1 might regulate gene transcription by sensing nuclear Ca^2+^ (Naranjo and Mellstrom, 2012; Nakamura et al., 2019). NCS-1 could control gene expression by indirect mechanisms, such as stimulation of Calmodulin kinase II, PI3/AKT-signaling (Petko et al., 2009; Nakamura et al., 2011), cAMP responsive elements (Souza et al., 2011), or DJ-1 (Xu et al., 2005; Takahashi-Niki et al., 2017). DJ-1 itself can translocate into the nucleus, particularly in response to metabolic stress, and regulates expression, e.g., of UCPs (Xu et al., 2018). Hence, the detected lower mRNA levels of UCP4 and UCP5 in NCS-1 KO mice might be secondary to the transcriptional downregulation of DJ-1. Lower levels of UCP4 and UCP5 were also described in SN tissue from DJ-1 KO mice (Guzman et al., 2010). Though the complex functions of DJ-1 are still not entirely clear, a support of mitochondrial function and reduction of metabolic stress is established (Biosa et al., 2017), and loss-of-function mutations in DJ-1 (PARK7) cause familial inherited PD (Bonifati et al., 2003).

Cav channel activity has also been linked to Ca^2+^ dependent regulation of gene expression (Gomez-Ospina et al., 2006; Barbado et al., 2009; Pinato et al., 2009). However, as mRNA levels of ENO2, UCP4, UCP5, and DJ-1 were all not altered in SN DA neurons of Cav2.3 KO mice, we conclude that their transcriptional downregulation in NCS-1 KO mice is rather not secondary to lower Cav2.3 levels. The higher ND1 mRNA and genomic levels in SN DA neurons from Cav2.3 KO might compensate for a reduced Ca^2+^ mediated stimulation of enzymes for ATP production. However, these are only theoretical speculations.

We found no evidence for transcriptional compensation of NCS-1 loss by KChip3. KChip3 (also named Calsenilin or DREAM) has overlapping functions with NCS-1 (Naranjo and Mellstrom, 2012; Burgoyne et al., 2019), and not only constitutes a beta subunit for Kv4.3 channels (An et al., 2000), that are involved in PD pathology (Subramaniam et al., 2014; Dragicevic et al., 2015), but it can also shuttle from the plasma-membrane to the nucleus, and act as a Ca^2+^ dependent transcription repressor by direct DNA binding (Carrion et al., 1999; Mellstrom and Naranjo, 2001; Gomez-Villafuertes et al., 2005; Mellstrom et al., 2014).

Possible compensation is generally important to consider, particularly for global knockouts. Both analyzed KO mouse strains have been already studied intensively and displayed clear phenotypes, in line with the described functions of NCS-1 and Cav2.3. The here studied NCS-1 KO mice showed decreased motivation, associated with lower dopamine release in the nucleus accumbens (Ng et al., 2016), and they are prone to gain weight and develop type 2 diabetes (Ratai et al., 2019). A different NCS-1 KO mouse strain, lacking exon 1 and resulting in disrupted NCS-1 protein, displayed an anxiety- and depression-like phenotype, reduced novelty-induced exploratory behavior (de Rezende et al., 2014), as well as reduced stress tolerance in cardiomyocytes due to dysfunctional mitochondrial detoxification and Ca^2+^ dependent pathways (Nakamura et al., 2016, 2019). Cav2.3-deficient mice display a mild cardiac, endocrine and neuronal phenotype (Pereverzev et al., 2005), assessed in four different KO mouse strains (Weiergraber et al., 2006). Mainly, they display slightly impaired insulin and somatostatin secretion (Jing et al., 2005; Zhang et al., 2007), mild cardiac arrhythmia (Weiergraber et al., 2005), and they are less prone to epilepsy (Weiergraber et al., 2007, 2010; Dibue-Adjei et al., 2017).

### NCS-1 – A Regulator of ATP Synthesis and Metabolic Stress in SN DA Neurons?

Our data suggest that NCS-1 activity in SN DA neurons is correlated with the expression of genes important for glycolytic and mitochondrial ATP production (ND1, ENO2), as well as of genes that control mitochondrial function and reduce metabolic stress (UCP4, UCP5, DJ-1). This might offer an explanation for a possible but not yet demonstrated neuroprotective function of NCS-1 for SN DA neurons: stimulation of ATP synthesis while at the same time controlling metabolic stress levels.

This conclusion would be in line with a reported NCS-1 stimulation of mitochondrial function and of Ca^2+^ dependent survival promotion in injured neurons in general (Angebault et al., 2018; Nakamura et al., 2019). However, it is important to note that we do not provide any functional data here to support this conclusion. We are currently addressing this issue by comparing respiration, ATP production capacity, and mitochondrial uncoupling in freshly-dissected vital SN slices from NCS-1 KO and wildtype mice via Seahorse XFe analysis.

Our findings are however well-complemented by a similar study in cardiomyocytes, at protein and functional levels (Nakamura et al., 2016). In cardiomyocytes of NCS-1 KO mice, the overall respiration and mitochondrial biogenesis was reduced, accompanied by a decreased functional expression of mitochondrial proteins. This phenotype could be rescued by NCS-1 overexpression, similar as described for respective mitochondrial dysfunction in fibroblasts from Wolfram Syndrome patients (Angebault et al., 2018). Furthermore, a reduced UCP-mediated proton leak in response to oxidative stress, accompanied with elevated mitochondrial oxidant stress, was described for cardiomyocytes of NCS-1 KO mice (Nakamura et al., 2016) – in line with the lower UCP4 and UCP5 mRNA levels that we report here for SN DA neurons.

Transcriptional downregulation of ND1, ENO2, and Cav2.3 might reflect a compensatory response to reduce stressful activity, related Ca^2+^ load, and ATP synthesis in SN DA neurons in the absence of protective NCS-1. However, these theoretical considerations would need to be experimentally addressed.

On a wider note, cell-specific stimulation of NCS-1 function (Mansilla et al., 2017) might offer a novel therapeutic strategy for combating metabolic stress and neurodegeneration. However, given the ubiquitous expression of NCS-1 and its multiple, complex, and still not fully understood functions, manipulation of this intricate network should be considered with caution.

## Ethics Statement

This study was carried out in accordance with the recommendations of the German Tierschutzgesetz, and the Regierungspräsidium Tübingen. The protocols were approved by the German Regierungspräsidium Tübingen (AZ 35/9185.81-3TV No. 1291, Reg. Nr. 0.147).

## Author Contributions

CS, ND, and JB carried out the molecular biology experiments. JD, JB, and CP performed the *in vivo* retrograde tracing. JD and JB contributed the UV-LMD of adult mice. OP provided NCS-1 KO mice. TS provided Cav2.3 KO mice. BL designed the study. BL, JD, JB, and CS wrote the manuscript. All authors revised the manuscript.

## Conflict of Interest

The authors declare that the research was conducted in the absence of any commercial or financial relationships that could be construed as a potential conflict of interest.
