# Supplementary material for: NCS-1 Deficiency Affects mRNA Levels of Genes Involved in Regulation of ATP Synthesis and Mitochondrial Stress in Highly Vulnerable Substantia nigra Dopaminergic Neurons
Source: Front Mol Neurosci. 2019 Nov 27;12:252. doi: 10.3389/fnmol.2019.00252 (PMC6890851; doi:10.3389/fnmol.2019.00252)
Supplement: Supplementary file 1 [file Data_Sheet_1.pdf]

Supplementary Information

Supplementary Figure S1, S2; Supplementary Tables S1 - S5

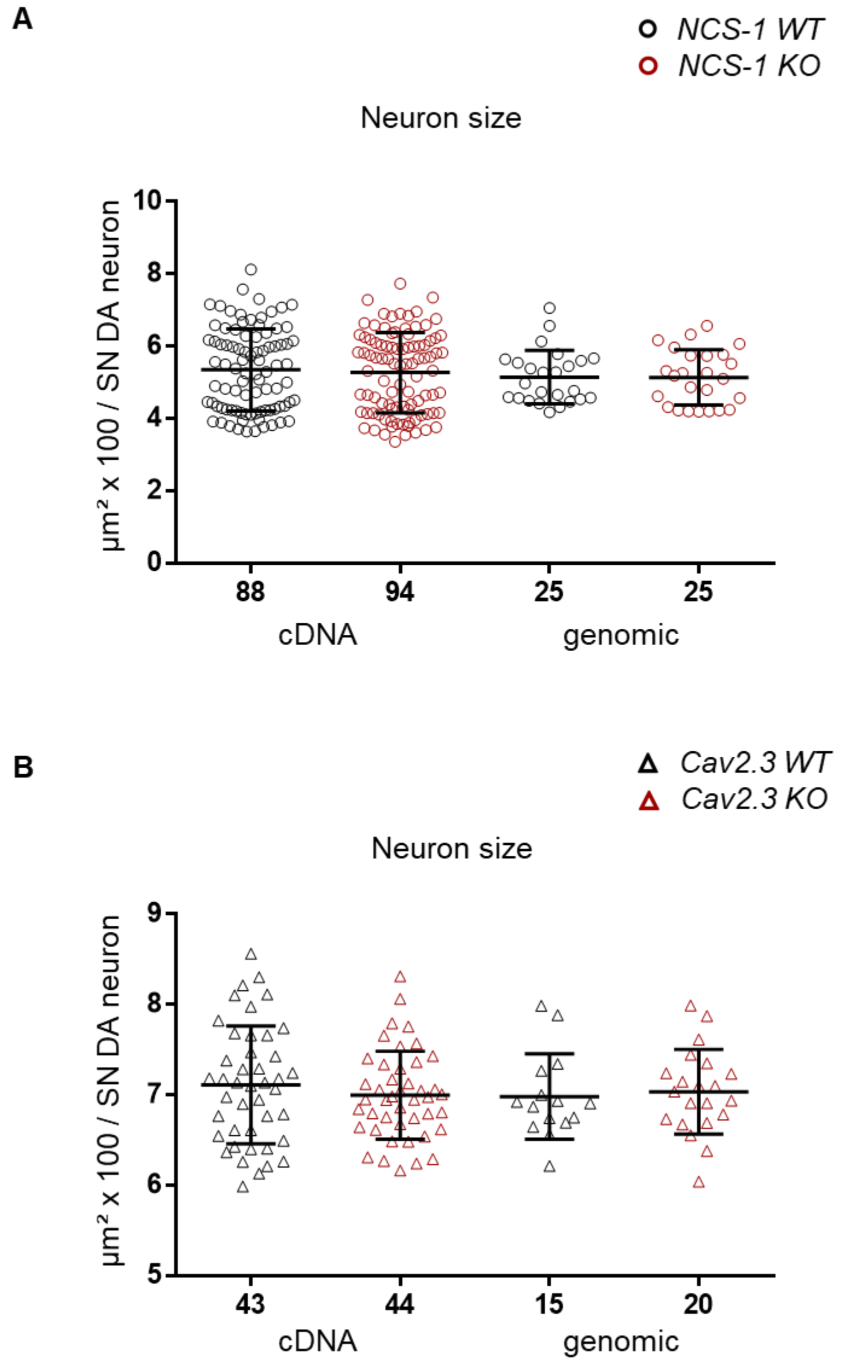

Figure S1 | SN DA neuron size in NCS-1 and Cav2.3 KO mice. Size [μm² x 100] of the individual laser-microdissected SN DA neurons from pools for cDNA and genomic DNA analysis in juvenile WT and NCS-1 KO mice **(A),** and from adult Cav2.3 WT and KO mice **(B)**. As both datasets show no significant difference, RT-qPCR data were normalized to respective lasered cell sizes. Data are given as scatter plots with mean ± SD. Number of analyzed cell pools (n) is given on the x-axis. Significances were tested by Mann-Whitney-U tests and t-tests. All data and statistics detailed in Supplementary **Table S2**.

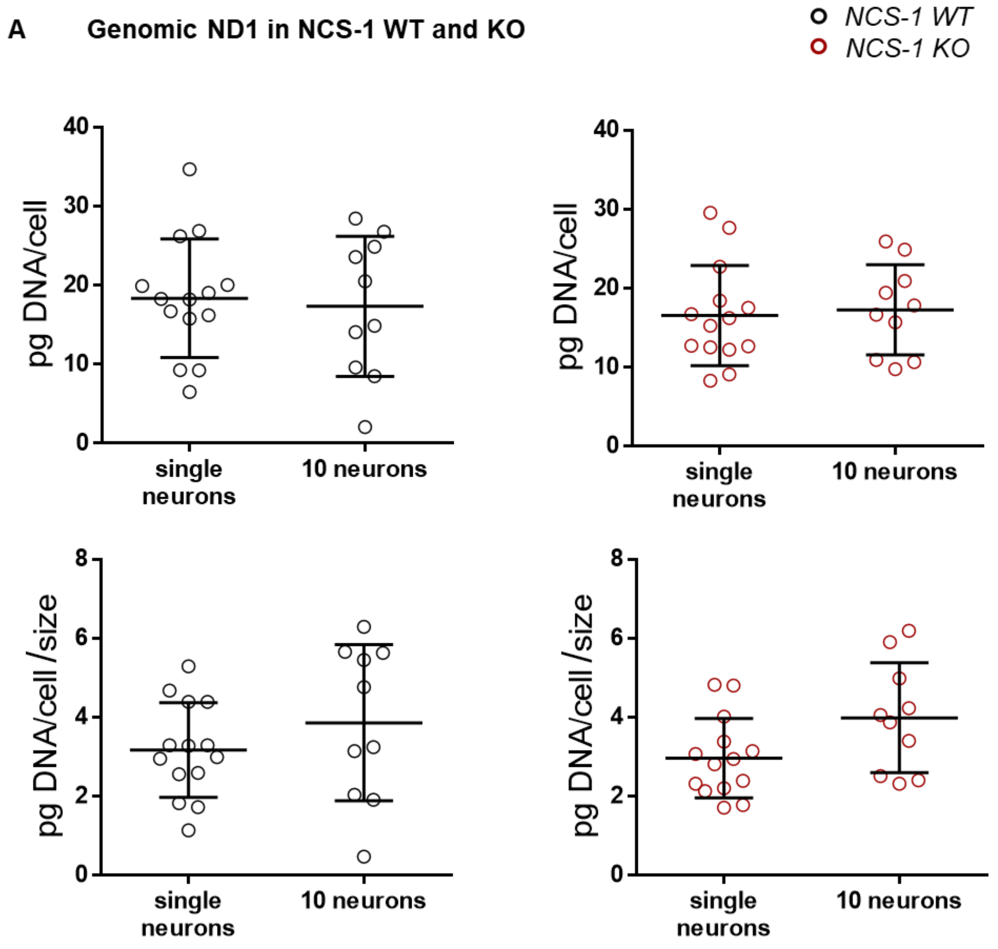
A
Genomic ND1 in NCS-1 WT and KO
NCS-1 WT
NCS-1 KO
pg DNA/cell
pg DNA/cell /size
single neurons
10 neurons

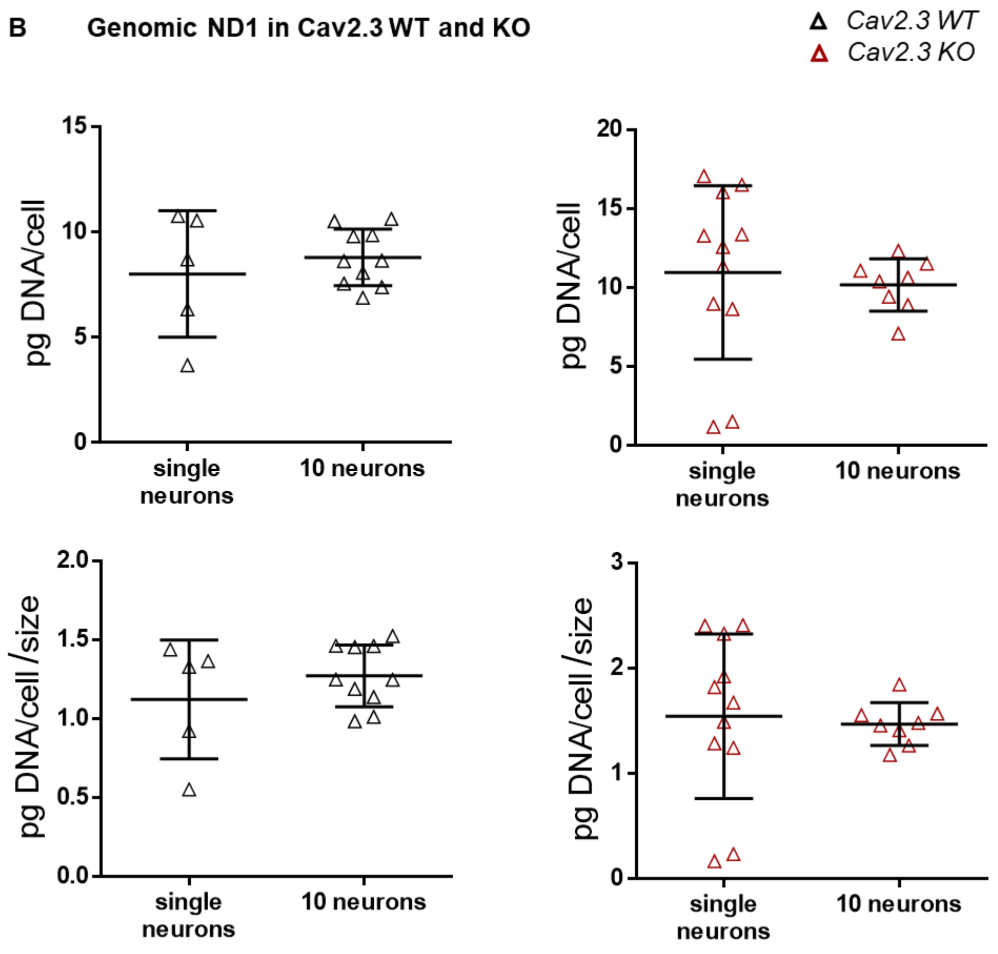
B
Genomic ND1 in Cav2.3 WT and KO
Cav2.3 WT
Cav2.3 KO
pg DNA/cell
pg DNA/cell /size
single neurons
10 neurons

Figure S2 | Genomic ND1 levels in individual SN DA neurons, derived from either single neurons or pools of 10 neurons. The determined genomic DNA amount / neuron was derived either from single SN DA neurons, or from 10 pooled neurons. As we detected no significant in mean copy number / individual SN DA neuron between both approaches, respective data sets were pooled. P-values according to Mann-Whitney-U tests: **(A)** NCS-1 WT, p=0.93; NCS-1 KO, p=0.71; NCS-1 WT/size, p=0.28; NCS-1 KO/size, p=0.63; **(B)** Cav2.3 WT, p>0.99; Cav2.3 KO, p=0.31; Cav2.3 WT/size, p= 0.44; Cav2.3 KO/size, p=0.44). Data are given as scatter plots with mean ± SD. Data and statistics for WT versus KO comparisons are detailed in Supplementary **Table S2**.

Table S1 | Primer information for Multiplex (outer) and nested (inner) PCRs of marker genes. F: forward; R: reverse.

Gene	Primer	Sequence (5`-3`)	Genbank accession no. (NCBI)	5`-Position	Amplicon size [bp]
Mouse Calbindin-d28k (CB)	F (outer)	CGCACTCTCAAACTAGCCG	M21531	87	891
	R (outer)	CAGCCTACTTCTTTATAGCGCA		977	
	F (inner)	GAGATCTGGCTTCATTTCGAC		167	440
	R (inner)	AGTTCCAGCTTTCCGTCATTA		606	
Mouse Glial fibrillary acidic protein (GFAP)	F (outer)	AGAACAACCTGGCTGCGTAT	K01347	407	786
	R (outer)	GCTCCTGCTTCGAGTCCTTA		1192	
	F (inner)	AGAAAGGTTGAATCGCTGGA		472	517
	R (inner)	CCAGGGCTAGCTTAACGTTG		988	
Mouse Glutamate decarboxylase (GAD65)	F (outer)	CATACGCAGACAGCACGTTT	NM_008078.1	166	905
	R (outer)	AAAAGATTCCATCGCCAGAG		1070	
	F (inner)	GGGATGTCAACTACGCGTTT		606	389
	R (inner)	CACAAATACAGGGGCGATCT		994	
Mouse Glutamate decarboxylase (GAD67)	F (outer)	TGACATCGACTGCCAATACC	Z49976	731	1105
	R (outer)	GGGTTAGAGATGACCATCCG		1835	
	F (inner)	CATATGAAATTGCACCCGTG		761	702
	R (inner)	CGGTGTCATAGGAGACGTCA		1462	
Mouse Tyrosine hydroxylase (TH)	F (outer)	CACCTGGAGTACTTTGTGCG	M69200	387	1139
	R (outer)	CCTGTGGGTGGTACCCTATG		1525	
	F (inner)	TGCACACAGTACATCCGTCA		936	377
	R (inner)	TCTGACACGAAGTACACCGG		1312	

Table S2 | Lasered neuron sizes, and qPCR results for ND1 (cDNA and genomic) and ENO2 (cDNA) for SN DA neurons from NCS-1 and Cav2.3 WT and respective KO mice. Data and statistics for graphs shown in **Figures S1, 2 and 4A**. (n) indicates the number of detected signals, from all tested cell pools (given in brackets), derived from (N) mice. P-values according to Mann-Whitney-U tests and significant differences are marked with (*). As similarity between WT and KO is essential for using neuron size as a normalization strategy, we additionally used t-tests in these cases after positive testing for Gaussian distribution. Respective p-values are marked with (^{T}). Both significance tests did not detect any alterations in neuron size between WT and KO. ND1 cDNA levels reflect cDNA + genomic DNA-derived signals, as the ND1 gene contains no intron.

	**NCS-1 WT**						**NCS-1 KO**						
	**mean**	**±SEM**	**±SD**	**median**	**n**	**N**	**mean**	**±SEM**	**±SD**	**median**	**n**	**N**	**p-value**
**Neuron size. cDNA** [µm^2 x 100]	5.34	0.12	1.14	5.33	88(88)	18	5.27	0.11	1.11	5.51	94 (94)	18	0.6195<br>0.6451^{T}
**Neuron size. genom.** [µm^2 x 100]	5.14	0.15	0.74	4.97	25(25)	3	5.13	0.15	0.76	5.18	25(25)	3	0.9272<br>0.9641^{T}
	**Relative qPCR results**												
**ND1 cDNA** [pg/cell]	41.00	2.29	21.40	37.58	87(88)	18	37.51	2.40	23.15	30.00	93(94)	18	0.1413
**ND1 genom single neurons** [pg/cell]	18.36	2.00	7.47	18.25	14(15)	3	16.54	1.70	6.35	15.73	14(15)	3	0.3580
**ND1 genom 10 neurons** [pg/cell]	17.34	2.80	8.85	17.69	10(10)	3	17.26	1.81	5.72	17.24	10(10)	3	0.9869
**ND1 genom. pooled data** [pg/cell]	17.94	1.61	7.91	18.25	24(25)	3	16.84	1.22	5.98	16.43	24(25)	3	0.5430
**ENO2 cDNA** [pg/cell]	63,98	4.95	46.12	41.14	87(88)	18	44.05	3.25	29.75	38.78	84(94)	18	**0.0274***
	**qPCR results, normalized to neuron sizes**												
**ND1 cDNA** [pg/µm^2 x 100]	7.99	0.40	3.66	7.85	87(88)	18	6.57	0.35	3.26	5.99	93(94)	18	**0.0152***
**ND1 genom single neurons** [pg/µm^2 x 100	3.17	0.32	1.20	3.14	14(15)	3	2.97	0.27	1.01	2.88	14(15)	3	0.5637
**ND1 genom 10 neurons** [pg/µm^2 x 100	3.86	0.62	1.98	4.00	10(10)	3	3.99	0.44	1.39	3.97	10(10)	3	0.8534
**ND1 genom. pooled data** [pg/µm^2 x 100]	3.45	0.32	1.57	3.26	24(25)	3	3.39	0.26	1.26	3.11	24(25)	3	0.8182
**ENO2** [pg/µm^2 x 100]	11.47	0.75	6.81	9.14	87(88)	18	8.24	0.53	4.71	7.47	84(94)	18	**0.0034***
	**Cav2.3 WT**						**Cav2.3 KO**						
	**mean**	**±SEM**	**±SD**	**median**	**n**	**N**	**mean**	**±SEM**	**±SD**	**median**	**n**	**N**	**p-value**
**Neuron size. cDNA** [µm^2 x 100]	7.11	0.10	0.65	7.13	43(43)	5	7.00	0.07	0.49	6.96	44(44)	5	0.4280<br>0.3596^{T}
**Neuron size. genom.** [µm^2 x 100]	6.98	0.12	0.47	6.90	15(15)	2	7.03	0.10	0.47	7.04	20(20)	3	0.5414<br>0.7381^{T}

	**Relative qPCR results**												
**ND1 cDNA** [pg/cell]	12.86	0.54	3.52	12.51	42(43)	5	14.52	0.58	3.85	13.81	44(44)	5	**0.0334***
**ND1 genom single neurons** [pg/cell]	8.01	1.34	3.00	8.71	5(10)	2	10.95	1.66	5.49	12.57	11(11)	3	0.1804
**ND1 genom 10 neurons** [pg/cell]	8.79	0.42	1.34	8.64	10(10)	2	10.17	0.59	1.66	10.51	8(9)	3	0.0676
**ND1 genom. pooled data** [pg/cell]	8.53	0.51	1.97	8.65	15(15)	2	10.62	0.97	4.24	11.08	19(20)	3	**0.0153***
**ENO2 cDNA** [pg/cell]	47.65	2.87	18.6	43.50	42(43)	5	49.70	3.24	21.47	49.12	44(44)	5	0.5501
	**qPCR results, normalized to neuron sizes**												
**ND1 cDNA** [pg/µm² x 100]	1.81	0.07	0.46	1.73	42(43)	5	2.07	0.07	0.49	1.94	44(44)	5	**0.0062***
**ND1 genom single neurons** [pg/µm² x 100	1.12	0.17	0.38	1.33	5(10)	2	1.54	0.24	0.78	1.67	11(11)	3	0.2212
**ND1 genom 10 neurons** [pg/µm² x 100	1.27	0.06	0.20	1.25	10(10)	2	1.47	0.07	0.20	1.47	8(9)	3	0.0676
**ND1 genom. pooled data** [pg/µm² x 100]	1.22	0.07	0.27	1.25	15(20)	2	1.51	0.60	0.60	1.49	19(20)	3	**0.0153***
**ENO2** [pg/µm² x 100]	6.68	0.37	2.38	6.48	42(43)	5	7.05	0.44	2.92	7.16	44(44)	5	0.5330

Table S3 | RT-qPCR Data for genes as indicated from SN DA neurons of NCS-1 WT and KO mice. Data and statistics for graphs shown in **Figure 3A/B/C**. (n) indicates the number of detected signals, from all tested cell pools (given in brackets), derived from (N) mice. P-values according to Mann-Whitney-U tests and significant differences are marked with (*).

	NCS-1 WT						NCS-1 KO						
	mean	±SEM	±SD	median	n	N	mean	±SEM	±SD	median	n	N	p-value
	**Relative qPCR results** [pg cDNA/cell]												
**UCP2**	402.9	132.2	373.9	270.1	8(15)	4	306.1	230.4	564.3	18.44	6(15)	4	0.5728
**UCP4**	117.9	13.28	39.83	115.6	9(9)	3	52.25	9.296	29.40	51.10	10(10)	3	**0.0004***
**UCP5**	48.29	12.11	36.34	35.25	9(9)	3	20.11	2.942	8.827	22.07	9(10)	3	**0.0188***
**MCU**	44.65	10.12	30.35	33.29	9(10)	3	40.69	10.68	28.26	35.58	7(10)	3	0.8371
**LETM1**	109.5	16.83	47.61	107.9	8(9)	4	71.02	12.82	38.47	58.23	9(9)	4	0.0927
**mNCX**	-	-	-	-	2(9)	3	-	-	-	-	0(10)	3	-
**DJ-1**	265.2	39.85	143.7	237.6	13(13)	6	116.9	14.78	49.02	102.6	11(14)	6	**0.0041***
**SNCA**	1152.0	167.8	474.6	1087.0	8(9)	3	1069.0	205.1	542.7	810.5	7(8)	4	0.4634
**PGC-1α**	46.84	10.89	34.44	37.66	10(10)	4	47.38	16.66	44.09	35.46	7(8)	4	0.9623
**GBA1**	52.21	5.251	15.75	53.01	9(10)	4	41.30	9.32	26.36	37.18	8(8)	4	0.1996
**Cav1.3**	68.37	12.92	38.76	52.97	9(10)	4	49.38	10.49	33.17	40.75	10(10)	3	0.1823
**Cav2.3**	706.3	54.82	245.2	680.2	20(20)	5	354.9	41.79	186.9	347.3	20(20)	4	**<0.0001***
**Cav3.1**	621.3	75.56	238.9	714.5	10(10)	4	493.8	89.36	282.6	378.6	10(10)	4	0.3527
**Kv4.3**	448.9	60.79	192.2	397.1	10(10)	3	487.2	127.7	383.0	354.0	9(10)	3	0.9048
**KChip3**	202.5	29.26	92.53	204.4	10(10)	3	203.9	43.89	138.8	147.6	10(10)	3	0.6842
	**qPCR results, normalized to neuron size** [pg cDNA/µm² x 100]												
**UCP2**	63.61	20.95	59.26	46.98	8(15)	4	46.74	33.80	82.79	2.68	6(15)	4	0.5728
**UCP4**	19.93	2.07	6.20	20.01	9(9)	3	8.82	1.58	5.01	8.62	10(10)	3	**0.0003***
**UCP5**	8.09	1.96	5.89	5.71	9(9)	3	3.37	0.49	1.47	3.84	9(10)	3	**0.0142***
**MCU**	7.49	1.84	5.53	5.13	9(10)	3	6.79	1.94	5.12	5.20	7(10)	3	0.9182
**LETM1**	25.86	4.45	12.59	23.77	8(9)	4	17.55	3.46	10.39	14.97	9(9)	4	0.0927
**mNCX**	-	-	-	-	2(9)	3	-	-	-	-	0(10)	3	-
**DJ-1**	57.80	3.53	32.25	46.86	13(13)	6	27.49	3.53	11.71	26.84	11(14)	6	**0.0129***
**SNCA**	296.2	47.15	133.4	271.8	8(9)	3	269.5	45.82	121.2	207.2	7(8)	4	0.4634
**PGC-1α**	11.08	2.43	7.68	9.07	10(10)	4	11.04	3.60	9.51	9.40	7(8)	4	0.8868
**GBA1**	12.64	1.30	3.89	13.51	9(10)	4	9.84	2.00	5.66	8.56	8(8)	4	0.1996
**Cav1.3**	9.51	1.53	4.60	8.33	9(10)	4	7.83	1.45	4.58	6.97	10(10)	3	0.3562
**Cav2.3**	106.0	9.15	40.90	93.50	20(20)	5	56.47	6.72	30.03	54.21	20(20)	4	**<0.0001***
**Cav3.1**	94.76	12.05	38.11	97.45	10(10)	4	77.82	14.89	47.09	53.46	10(10)	4	0.4359
**Kv4.3**	70.23	10.21	32.28	63.33	10(10)	3	78.05	20.22	60.65	59.29	9(10)	3	0.9682
**KChip3**	31.77	5.11	16.16	28.68	10(10)	3	33.53	7.56	23.90	23.58	10(10)	3	0.8534

Table S4 | RT-qPCR Data for genes as indicated from SN DA neurons of Cav2.3 WT and KO mice. Data and statistics for graphs shown in **Figure 4B**. (n) indicates the number of detected qPCR signals, from all tested cell pools (given in brackets), derived from (N) mice. P-values according to Mann-Whitney-U tests.

	**Cav2.3 WT**						**Cav2.3 KO**						
	**mean**	**±SEM**	**±SD**	**median**	**n**	**N**	**mean**	**±SEM**	**±SD**	**median**	**n**	**N**	**p-value**
	**Relative qPCR results** [pg cDNA/cell]												
**DJ-1**	68.98	8.14	25.75	60.8	10(10)	3	61.81	10.8	34.16	56.61	10(10)	3	0.5787
**UCP4**	39.81	3.88	12.27	40.24	10(10)	3	53.38	9.04	28.59	53.21	10(10)	3	0.2475
**UCP5**	35.53	4.59	18.37	33.28	16(16)	4	41.14	4.43	17.71	38.16	16(16)	4	0.2871
	**qPCR results, normalized to neuron size** [pg cDNA/µm² x 100]												
**DJ-1**	9.50	0.89	2.81	8.50	10(10)	3	8.89	1.50	4.73	8.11	10(10)	3	0.6305
**UCP4**	5.88	0.60	1.91	5.99	10(10)	3	7.55	1.24	3.92	7.62	10(10)	3	0.2799
**UCP5**	5.16	0.69	2.77	4.65	16(16)	4	5.87	0.63	2.51	5.39	16(16)	4	0.3414

Table S5 | Relation of significantly different RT-qPCR data from Table S3 to that of ND1 or ENO2 in for SN DA neurons from NCS-1 WT and KO mice. Data and statistics for graphs shown in **Figure 5**. pg cDNA/µm² x 100 data is normalized to pg cDNA/cell of ND1 or respective ENO2. (n) indicates the number of detected qPCR signals, from all tested cell pools (given in brackets), derived from (N) mice. P-values according to Mann-Whitney-U tests and significant differences are marked with (*).

	NCS-1 WT						NCS-1 KO						
	mean	±SEM	±SD	median	n	N	mean	±SEM	±SD	median	n	N	p-value
	Relative qPCR results, normalized to neuron size and ND1												
**UCP4**	0.44	0.03	0.08	0.45	9(9)	3	0.25	0.03	0.11	0.28	10(10)	3	**0.0003***
**UCP5**	0.16	0.02	0.07	0.13	9(9)	3	0.10	0.02	0.05	0.08	9(10)	3	**0.0503***
**DJ-1**	1.44	0.10	0.35	1.42	13(13)	6	1.28	0.09	0.28	1.28	9(14)	6	0.2921
**Cav2.3**	2.21	0.17	0.75	2.00	20(20)	5	1.24	0.13	0.55	1.15	19(20)	4	**<0.0001***
**ENO2**	0.28	0.02	0.15	0.26	86(88)	18	0.28	0.01	0.14	0.27	87(94)	18	0.9722
	Relative qPCR results, normalized to neuron size and ENO2												
**UCP4**	0.21	0.01	0.04	0.21	9(9)	3	0.14	0.02	0.07	0.13	10(10)	3	**0.0350***
**UCP5**	0.08	0.01	0.04	0.06	9(9)	3	0.06	0.01	0.04	0.05	9(10)	3	0.1615
**DJ-1**	1.43	0.25	0.86	1.22	12(13)	6	1.92	0.60	1.80	1.14	9(14)	6	0.9170
**Cav2.3**	1.05	0.11	0.50	0.82	20(20)	5	0.84	0.10	0.42	0.71	18(20)	4	0.1957
**ND1**	0.19	0.02	0.15	0.11	84(88)	18	0.15	0.01	0.09	0.11	81(94)	18	0.5682
